# The health of mothers caring for a child with a disability: a longitudinal study

**DOI:** 10.1186/s12905-023-02798-y

**Published:** 2023-11-30

**Authors:** Idunn Brekke, Andreea Alecu

**Affiliations:** 1 Department of Childhood and Families, Division of Mental and Physical Health, Norwegian Institute of Public Health, PO Box 222, Skøyen, Oslo, N-0213 Norway; 2 Consumption Research Norway, Oslo Metropolitan University, Oslo, Norway

**Keywords:** Maternal health, Child disability, Longitudinal data, Mental health, Caring

## Abstract

**Background:**

Raising a child with disabilities requires a significant parental investment that is greater than that required by typically developing children. Previous studies have shown that parents caring for a child with a disability experience a range of health problems, particularly the mothers. However, few of these studies have controlled for maternal health prior to birth.

**Methods:**

This study used a sample from the Norwegian administrative register that comprised all children born between 2009 and 2015. We followed the mothers and their children for 11 years, between 2009 and 2019. The outcome variable was the mothers’ physical and mental health, which was assessed using specific ICD-10 diagnoses recorded in the Norwegian Patient Register (NPR). The data included information on the mothers’ health before and after the birth of their first child, enabling us to control for maternal health prior to birth in our analysis, in addition to socio-demographic characteristics. The analyses of maternal health were performed using multiple logistic regression, and the results are presented on both a relative scale (odds ratio [OR]) and an absolute scale (average marginal effect [AME]), both with 95% confidence intervals.

**Results:**

Mothers caring for a child with a disability have higher odds of having a diagnosis of a musculoskeletal disorder, depression, anxiety, sleeping disorder or migraines than mothers of children without a disability. The differences between the two groups of mothers decrease after adjusting for the characteristics of the children, mothers and families, but remain significant for musculoskeletal disorder, depression, anxiety and sleeping disorder, although the absolute differences are modest.

**Conclusion:**

The findings suggest that mothers caring for a child with a disability are more likely to have health problems than mothers of children without a disability after controlling for maternal health prior to birth. Providing more support for mothers of children with a disability might help to improve their health.

**Supplementary Information:**

The online version contains supplementary material available at 10.1186/s12905-023-02798-y.

## Introduction

Raising a child with disabilities requires a significant parental investment that is greater than that required by typically developing children and can include specialised medical care, numerous medical visits and coordinated support from multiple siloed agencies [1]. Mothers are often the primary caregivers and take on additional roles, such as case manager, therapist and teacher [2]. The additional costs associated with caring for a child with disability [3] also increase the risk of economic hardship for families [4], and the demands of care and coordinating help and support from different agencies can be time-consuming and stressful, adversely affecting the employment [5] and health of the parents, particularly the mothers [6, 7].

Maternal health is crucial not only for the mothers themselves, but also for the child’s development [8]. For example, children exposed to maternal depression have a higher risk of developmental difficulties than the children of mothers without depression [9]. The health of mothers caring for a child with a disability therefore requires research attention to help us better understand the implications of caring for a child with special needs.

There is extensive literature on the health of parents who care for children with disabilities [10, 11], which generally shows that they have more health problems than the parents of children without disabilities. A growing body of literature has shown an association between parenting a child with disabilities and depression, anxiety [12–16], sleeping disorders [6, 17], musculoskeletal problems [18] and physical health problems, such as cardiovascular disease and backpain [15]. Adverse health effects appear to be more pronounced among mothers than fathers [19], while studies with non-significant findings are limited (see [20, 21] for examples).

However, many studies on the topic rely on cross-sectional survey analysis using small samples or non-representative groups, and there is limited longitudinal research with large samples that uses administrative register data. Marquis et al. [12] used population-level administrative data, confirming that the parents of children with developmental disabilities have more depressive symptoms, but their study did not account for parental health prior to birth; indeed, little is known about the changes in the health of parents of children with disabilities before and after childbirth. One such study is Arim et al. [22], who used longitudinal administrative health data, finding that the mothers of children with neurodevelopmental disabilities (NDD) were more likely to have chronic health conditions and higher healthcare service utilisation than parents of children without NDD and also that this pattern was present prior to the child’s birth.

The aim of this study was to examine the association between child disability and five indicators of maternal health. We used 11 years of high-quality, longitudinal, Norwegian population administrative register data to observe mothers and their children. The data included information on the mothers’ health before and after the birth of their first child, enabling us to control for maternal health prior to birth in our analysis, in addition to socio-demographic characteristics.

## Methods

### Study design and sample

Our sample consists of children born in Norway between 2009 and 2015, and we observed the mothers and their first-born children in a longitudinal person–year format between 2009 and 2019 (n = 1,594,900). Children and mothers who died within the observation period are removed out from the analyses. We used several data sources to link the mothers with their children, including the Central Population Register, the Historical Event Database (FD-trygd) and the National Educational Database, which provide information on income, welfare benefits, employment, education and demographics. Children who need long-term private care and supervision because of a medical condition are entitled to financial assistance from the Norwegian Labour and Welfare Administration [23], and children with disabilities can therefore be identified using information on assistance allowances included in FD-trygd.

Assistance allowances are a non-means-tested cash benefit adjusted to the severity of increased care needs, for which parents need to file an application. The care needs must last for two to three years or more, and there are four levels of benefit payments, reflecting mild to severe care needs, with the overall workload of the person providing the care/supervision being the determining factor [23].

Socio-demographic and child disability data were linked to the Norwegian Patient Register (NPR) containing information on the mothers’ physical and mental health. The NPR is an administrative database of records reported by all government-owned hospitals, outpatient clinics and private health clinics that receive governmental reimbursement. The register contains individual information about all treatments received from specialist healthcare services. The use of encrypted national ID numbers in the NPR was started in 2008, enabling linkage to other national registers. Diagnostic codes using the World Health Organization’s International Classification of Diseases, version 10 (ICD-10), are recorded in the NPR.

This study was approved by the Regional Committee for Medical Research Ethics in South-East Norway (116,474). The Norwegian Data Protection Authority granted permission to access all the databases and records without written consent from the study’s participants.

### Measures

#### Primary outcomes

Our outcome measure was mothers’ physical and mental health, assessed using specific ICD-10 diagnoses in the NPR: musculoskeletal disorders (ICD-10: M00–M99, excluding juvenile diagnoses), anxiety (ICD-10: F40, F41), depression (ICD-10: F32, F33), migraines (ICD-10: G43) and sleep disorders (ICD-10: G47, F51). The variable used in the analysis was coded as 1 if the mother was diagnosed with one of these diagnoses at any time after the child was born and 0 otherwise.

#### Child disability

Children who receive assistance allowances were classified as children with a disability. The assistance allowance is paid at four different rates, reflecting mild to severe care needs, and we used this payment level as a proxy for the severity of the child’s disability, ranging from 1 to 4. We equated payment level 1 with a mild disability (coded as 1) and payment levels 2 to 4 with a complex disability (coded as 2); the reference group was non-disabled children—those not reported as receiving an assistance allowance (coded as 0).

#### Explanatory variables

We included variables that previous research has shown affect maternal health [10] and that were available from our data sources. The age of the mother at birth is measured in years, but to allow for non-linearity, we included age-squared in the model. The mother’s region of birth was classified as (1) Norway; (2) EU/EEA, USA, Canada and Australia; or (3) Asia, Africa and Latin America. The mother’s highest completed educational level was measured in years. We included the EU equivalised disposable household income, which is calculated by dividing the household’s total income by its equivalent size using the modified EU equivalence scale, which attributes a weighting to all household members. The household income is measured in quintiles, with lowest being the reference category.

Maternal employment was coded as 1 if the mother was employed one year prior to the birth and 0 otherwise. Parental divorce was coded as 1 if divorced or separated and 0 if married or living with a partner. The number of children in the household was indicated by three dummy variables: (1) single child, (2) two children and (3) three or more children. We also controlled for maternal health prior to the child’s birth, which was coded as 1 if the mother had one of the selected diagnoses before the child was born and 0 otherwise. Finally, we included the age of the child, the age of the child squared and the sex of the child. All the variables included in the model were time-varying, except the mother’s region of birth.

### Statistical analyses

Descriptive analyses are presented with means (SD) and proportions (%). The analyses of maternal health were performed using multiple logistic regression, and the results are presented on both a relative scale (odds ratio [OR]) and an absolute scale (average marginal effect [AME]), both with 95% confidence intervals. Unlike ORs, marginal effects are not influenced by the amount of unexplained variance in a model and can be compared across models and samples [24].

We examined five classes of physical and mental health—musculoskeletal disorders, depression, anxiety, migraines and sleeping disorders—and contrasted mothers caring for a child with a disability to a control group of mothers caring for a child without a disability. We present results separately for the five health outcomes, and all the analyses are presented as both unadjusted and adjusted estimates (i.e., both before and after controlling for characteristics of the children, mothers and families). The variance inflation factor (VIF) was used to determine if the variance of the estimated regression coefficient was inflated due to collinearity among the independent variables in the regression models. If we remove the quadratic terms for age (i.e. only include child’s and respectively mother’s age) in the model, the average VIF is 1.75. which showed that multicollinearity was not a main concern in this study given Midi & Bagheri [25] who propose that a VIF score greater than 10 indicates the presence of multicollinearity. Robust standard errors were used, and the statistical analysis was performed using STATA® 17. Some information was missing regarding educational level (4% of participants), household income (6%) and employment prior to birth (18%). Those with missing information were included as a separate category in the analyses. Due to the size of the dataset, the significance level was set at p < 0.01 for the entry of each variable into the model and also as the criterion for the variable to be retained.

## Results

Table 1 shows the means, SDs and proportions (%) for all the independent variables used in the regression analyses; they are presented separately for the mothers of children without disabilities, with mild disabilities and with complex disabilities.

**Table 1:** Descriptive statistics of variables used in the analysis, n = person-years

Variable	Without disability, n = 1,574,328	Mild disability, n = 6,343	Complex disability, n = 14,229
Musculoskeletal disorders (n (%))	50,283 (3.2%)	329 (5.2%)	736 (5.2%)
Depression (n (%))	23,672 (1.5%)	139 (2.2%)	339 (2.4%)
Anxiety (n (%))	21,234 (1.3%)	119 (1.9%)	280 (2.0%)
Migraine (n (%))	7,561 (0.5%)	52 (0.8%)	123 (0.9%)
Sleeping disorder (n (%))	3,161 (0.2%)	34 (0.5%)	60 (0.4%)
Have had diagnosis prior to birth			
None (n (%))	1,428,802 (91%)	5,573 (88%)	12,866 (90%)
Yes, at least one (n (%))	145,526 (9.2%)	770 (12%)	1,363 (9.6%)
Year of education, mother, mean (SD)	16.0 (4.08)	15.3 (4.29)	15.4 (4.19)
Household income quintile			
Lowest (n (%))	293,595 (19%)	1,154 (18%)	2,36 (17%)
Second (n (%))	266,492 (17%)	1,289 (20%)	2,617 (18%)
Third (n (%))	292,679 (19%)	1,364 (22%)	2,879 (20%)
Fourth (n (%))	318,568 (20%)	1,343 (21%)	3,306 (23%)
Highest (n (%))	310,451 (20%)	1,147 (18%)	2,930 (21%)
Number of children			
Single child (n (%))	297,200 (19%)	1,144 (18%)	3,182 (22%)
Two children (n (%))	831,425 (53%)	3,192 (50%)	6,699 (47%)
Three or more children (n (%))	445,703 (28%)	2,007 (32%)	4,348 (31%)
Employment status			
Unemployed (n (%))	297,664 (19%)	1,474 (23%)	3,751 (26%)
Employed (n (%))	1,017,859 (65%)	4,071 (64%)	8,590 (60%)
Marital status			
Married/partner (n (%))	1,411,309 (90%)	6,004 (95%)	13,111 (92%)
Divorced/Separated (n (%))	56,223 (3.6%)	300 (4.7%)	995 (7.0%)
Region of birth			
Norway (n (%))	1,087,549 (69%)	4,639 (73%)	9,906 (70%)
Europe, North America, Australia (n (%))	210,763 (13%)	564 (8.9%)	1,354 (9.5%)
Africa, Asia, Latin America (n (%))	276,003 (18%)	1,140 (18%)	2,969 (21%)
Age at birth, mean (SD)	29.30 (5.16)	29.01 (5.44)	29.12 (5.60)
Child age, mean (SD)	3.78 (2.70)	4.67 (2.54)	5.90 (2.37)
Child sex			
Boys (n (%))	807,617 (51%)	3,939 (62%)	9,162 (64%)
Girls (n (%))	766,711 (49%)	2,404 (38%)	5,067 (36%)

### Maternal health

**Fig. 1:**
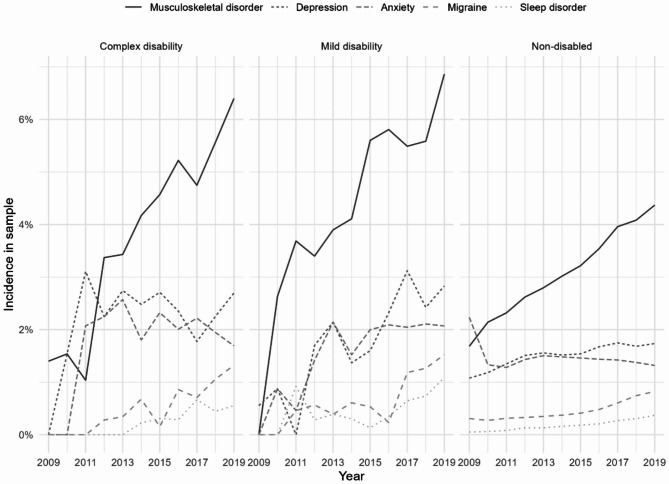
Percent of the mothers registered with anxiety, depression, migraine, musculoskeletal disorders or sleeping disorders in the given year by the child’s disability, n = person-years Abbreviations: NDD—children with neurodevelopmental disabilities, NPR—the Norwegian Patient Register, ICD-10—International Classification of Diseases, version 10, SD—Standard deviation, OR—Oddsratio, AME—average marginal effects

Figure 1 shows the percentages of the mothers diagnosed with anxiety, depression, migraines, musculoskeletal disorders or sleeping disorders in the given year according to their child’s disability. Musculoskeletal disorders were the most widespread diagnosis, followed by depression, anxiety, sleeping disorders and migraines. Although the prevalences of the five health conditions were low for all the mothers, the figure shows that mothers caring for a child with a disability were more likely to have one of the diagnoses than mothers of children without a disability.

Table 2 shows unadjusted and adjusted results obtained from the logistic regression models for the associations between child disabilities and the five maternal health diagnoses. In the unadjusted model, mothers caring for a child with a disability had significantly higher odds of having one of the five health problems than mothers caring for a child with no disability. Adjusting for the characteristics of the children, mothers and families slightly decreases the differences between the two groups, but even with all the other variables held constant, mothers caring for a child with a disability still had significantly higher odds of having a health problem after birth than mothers of children without a disability, except from migraine, which turn out to be insignificant in the adjusted model.

The adjusted results show that mothers of a child with a complex disability had 1.19 times higher odds of having a musculoskeletal diagnosis than mothers of children without a disability, which equates to a difference of 0.6% points (pp) on an absolute scale (see Online Supplementary Appendix A). The results for depression were 1.25 times higher odds (0.4 pp), for anxiety were 1.30 times (0.4 pp), for sleeping disorders were 1.27 times (0.06 pp) and for migraines were 1.18 times (0.09 pp), however the OR for migraine was not significant. Very similar results were found for mothers caring for a child with a mild disability (Table 2). Overall, we found that caring for a child with a disability increases the odds of having one of the selected health problems after birth, although the (absolute) magnitude of the associations seems modest.

Table 2 also shows that mothers who had health problems prior to the birth had substantially higher odds of having one of the five diagnoses afterwards, but having a higher education decreased the odds of having musculoskeletal disorders, depression, anxiety, sleeping disorder, or migraine. Being in the highest income quintile was associated with decreased odds of having one of the five diagnoses. Going through a divorce during the observation period increased the odds of having musculoskeletal diagnosis and depression after the birth. Having more than one child, being born outside Norway and being employed prior to the birth decreased the odds of having a diagnosis. Moreover, the odds of having musculoskeletal disorders, depression, sleeping disorders or migraine increased with the child’s age.

**Table 2:** Predictors of maternal health problems, Odds ratios (ORs) and 95% confidence intervals in brackets

	(1)	(2)	(3)	(4)	(5)	(6)	(7)	(8)	(9)	(10)
	Musculoskeletal disorders	Musculoskeletal disorders adjusted	Depression	Depression adjusted	Anxiety	Anxiety adjusted	Sleeping disorder	Sleeping disorder adjusted	Migraine	Migraine adjusted
**Child disability**, no disability (ref.)										
Mild disability	1.658^***^	1.298^***^	1.468^***^	1.170	1.398^***^	1.198	2.679^***^	1.970^***^	1.713^***^	1.312
	[1.483,1.853]	[1.157,1.455]	[1.240,1.737]	[0.986,1.388]	[1.166,1.677]	[0.997,1.438]	[1.909,3.759]	[1.402,2.768]	[1.303,2.252]	[0.992,1.736]
Complex disability	1.653^***^	1.187^***^	1.599^***^	1.252^***^	1.468^***^	1.307^***^	2.105^***^	1.273	1.807^***^	1.179
	[1.534,1.782]	[1.099,1.282]	[1.434,1.782]	[1.121,1.398]	[1.303,1.654]	[1.156,1.477]	[1.629,2.719]	[0.984,1.648]	[1.511,2.161]	[0.977,1.423]
**Have had diagnosis prior to birth**: no (ref.)										
Yes, at least one		3.180^***^		3.278^***^		2.629^***^		2.439^***^		2.941^***^
		[3.111,3.250]		[3.181,3.378]		[2.545,2.716]		[2.234,2.664]		[2.783,3.107]
**Year of education, mother**		0.962^***^		0.990^***^		0.968^***^		0.966^***^		0.991^**^
		[0.959,0.964]		[0.986,0.993]		[0.964,0.972]		[0.956,0.975]		[0.985,0.998]
**Household income** lowest quintile (ref.)										
Second		1.255^***^		1.006		1.034		0.992		1.193^***^
		[1.216,1.295]		[0.966,1.047]		[0.990,1.080]		[0.882,1.116]		[1.100,1.294]
Third		1.261^***^		0.877^***^		0.975		0.996		1.236^***^
		[1.222,1.302]		[0.841,0.915]		[0.932,1.020]		[0.885,1.121]		[1.139,1.342]
Fourth		1.192^***^		0.759^***^		0.901^***^		0.891		1.315^***^
		[1.153,1.232]		[0.725,0.793]		[0.859,0.945]		[0.788,1.007]		[1.210,1.429]
Highest		1.038^*^		0.617^***^		0.802^***^		0.678^***^		1.261^***^
		[1.003,1.075]		[0.588,0.649]		[0.762,0.845]		[0.594,0.773]		[1.156,1.377]
**Number of children**, Single child (ref.)										
Two children		0.879^***^		0.712^***^		0.924^***^		0.698^***^		0.882^***^
		[0.857,0.901]		[0.689,0.737]		[0.889,0.959]		[0.639,0.763]		[0.827,0.940]
Three or more children		0.820^***^		0.631^***^		0.840^***^		0.537^***^		0.804^***^
		[0.798,0.843]		[0.607,0.655]		[0.806,0.876]		[0.484,0.595]		[0.749,0.863]
**Employment status**, non-employed (ref.)										
Employed		0.824^***^		0.651^***^		0.590^***^		0.733^***^		0.792^***^
		[0.805,0.844]		[0.630,0.672]		[0.570,0.610]		[0.671,0.802]		[0.746,0.841]
**Marital status**, Married/partner (ref.)										
Divorced/Separated		1.119^***^		1.664^***^		1.056		1.098		1.115
		[1.073,1.167]		[1.577,1.755]		[0.982,1.136]		[0.947,1.274]		[0.999,1.245]
**Region of birth**, Norway (ref.)										
Europe, North America, Australia		0.758^***^		0.677^***^		0.548^***^		0.677^***^		0.657^***^
		[0.731,0.785]		[0.643,0.713]		[0.517,0.582]		[0.592,0.775]		[0.598,0.723]
Africa, Asia, Latin America		0.770^***^		0.519^***^		0.357^***^		0.544^***^		0.634^***^
		[0.748,0.793]		[0.496,0.544]		[0.337,0.378]		[0.481,0.616]		[0.584,0.689]
**Age at birth**		0.968^***^		0.901^***^		1.021		0.911^***^		0.948^*^
		[0.954,0.983]		[0.883,0.921]		[0.997,1.046]		[0.865,0.959]		[0.911,0.988]
Age at birth squared		1.001^***^		1.002^***^		1.000^*^		1.002^***^		1.000
		[1.001,1.001]		[1.001,1.002]		[0.999,1.000]		[1.001,1.003]		[1.000,1.001]
**Child age**		1.152^***^		1.099^***^		0.990		1.425^***^		1.149^***^
		[1.138,1.165]		[1.082,1.117]		[0.974,1.007]		[1.356,1.498]		[1.116,1.183]
**Child sex**, boys (ref.)		0.998^***^		0.996^***^		1.001		0.986^***^		1.000
		[0.996,0.999]		[0.995,0.998]		[0.999,1.003]		[0.981,0.990]		[0.997,1.003]
Girls		1.003		0.938^***^		0.965^*^		0.968		0.999
		[0.985,1.021]		[0.914,0.963]		[0.939,0.992]		[0.903,1.038]		[0.955,1.045]
n (person-years)	1,594,900	1,490,196	1,594,900	1,490,196	1,594,900	1,490,196	1,594,900	1,490,196	1,594,900	1,490,196
*AIC*	453670.3	414069.0	250249.4	229657.8	228989.0	212674.4	46785.0	43345.6	97838.5	91294.8
*BIC*	453707.1	414349.9	250286.3	229938.7	229025.8	212955.3	46821.9	43626.5	97875.3	91575.8

^*^p < 0.05, ^**^p < 0.01, ^***^p < 0.001

## Discussion and conclusion

Raising a child with disabilities requires a significant parental investment that is greater than that required by typically developing children. Despite numerous studies showing an adverse association between caring for a child with a disability and maternal health ([e.g.10], for example), previous research has several methodological limitations, including small sample sizes, the use of cross-sectional survey data and bias caused by omitted variables. The findings of associations between child disability and maternal health might therefore be spurious and a result of unobserved confounders affecting both child disability and maternal health, and we could thus not be certain that the increased health problems among mothers with a child with a disability were caused by the caring burden. Arim et al. [22] found that mothers of children with NDD are more likely to have health problems than mothers who have a child without NDD, but this pattern was also found prior to the birth, which underlines the importance of controlling for previous maternal health. Moreover, previous studies on this subject are often based on subjective measures of health provided by survey data and are thus subject to measurement error.

We overcame these limitations in several ways. Our rich longitudinal data allowed us to control for a broad array of time-varying and time-invariant covariates to deal with potentially confounding factors. Most importantly, our data allowed us to control for maternal health prior to the birth and to focus on the first-born child, reducing the risk of omitted-variable bias. We also used population administrative register data, which is less prone to sampling error and selection bias [26].

The purpose of this study was to examine the association between child disability and maternal health, and in the unadjusted model we found that caring for a child with a disability increases the odds of having musculoskeletal disorders, depression, anxiety, sleeping disorders and migraines. To examine whether the association between child disability and maternal health is confounded by differences in mothers’ characteristics prior to the child’s birth, we used models to control for prior maternal health and a range of characteristics of the children, mothers and families. The results from the adjusted model confirm the results from the unadjusted model, however including the covariates in the adjusted model slightly reduces the strength of the association between child disability and the maternal health indicators, and migraine did not reach statistical significance. Nevertheless, we still found significant associations between child disability and four of the maternal health categories examined.

Our results are in line with previous research that also shows that mothers caring for a child with a disability are more likely to have health problems than mothers of a child without a disability (for examples, [see e.g.10, 11]), although the strength of these associations can further vary with the child’s diagnosis and the type of maternal health problem [11]. The ORs estimated in the current study appear similar to the ORs reported in Marquis et al. [12]. Reporting guidelines like STROBE recommend presenting both relative and absolute measures [27], so we have presented the results on both a relative scale (ORs) and an absolute scale (AMEs), which has implications for the interpretation of the results because they provide different perspectives [28]. For example, the relative differences in the present study seem more impactful than the absolute differences.

Most of the studies in this field have used self-reported measures of parental health and have found sizeable differences between parents caring for a child with a disability and parents caring for a child without a disability. Our data was restricted to mothers who have received specialist healthcare treatment, which means that we captured only the more severe health conditions. However, many health problems are not reported to specialist healthcare services, and the literature reports that mothers caring for a child with a disability have a broad range of health problems that may not be registered by specialist healthcare. Our data, therefore, only shows the tip of the iceberg of health problems in this group of mothers, but even so, there is still a clear pattern. Mothers caring for a child with a disability are significantly more likely to have musculoskeletal disorders, depression, anxiety and sleeping disorders than mothers of children without a disability.

The strengths of this study are the large sample size, longitudinal design and wide range of socio-demographic, health and work-related pre- and post-birth data, in contrast to the cross-sectional studies that have dominated this area of research. We used administrative longitudinal population data that is not significantly affected by participant attrition, and our study is one of the few in this field to control for maternal health prior to birth, enabling us to rule out an important confounder. An additional strength is the inclusion of data on the severity of the child’s condition. However, we found little support for differences in health between the mothers of children with mild and complex disabilities. Children with complex disabilities are entitled to more economic support and can apply for other forms of assistance, such as respite care and user-controlled personal assistance, which may partly explain this finding [29].

Nevertheless, there are limitations. We identified children with a disability only if they were administratively recognised as such and received assistance allowance, which does not encompass all children with a disability and may underrepresent those with less severe conditions. The data also only included mothers who received specialist healthcare treatment and, again, may not have captured less severe conditions, so differences in health problems between mothers of children with disabilities and mothers of children without disabilities might be underestimated. Despite these limitations, our study shows that mothers caring for a child with a disability are more likely to have health problems than mothers of children without a disability after controlling for maternal health prior to birth, although the differences are modest.

### Electronic supplementary material

Below is the link to the electronic supplementary material.

Supplementary Material 1

## Data Availability

The data used in this study are available from Statistics Norway and the Norwegian Directorate of Health, but they are not publicly accessible because they were used under license for this study. The data may, however, be made available upon request to the first author granted permission is given by Statistics Norway and the Norwegian Directorate of Health.
