# Supplementary material for: The health of mothers caring for a child with a disability: a longitudinal study
Source: BMC Womens Health. 2023 Nov 30;23:639. doi: 10.1186/s12905-023-02798-y (PMC10688054; doi:10.1186/s12905-023-02798-y)
Supplement: Supplementary file 1 — Supplementary Material 1 [file 12905_2023_2798_MOESM1_ESM.docx]

**ELECTRONIC SUPPLEMENTAL TABLE**

**The health of mothers caring for a child with a disability: a longitudinal study**

**Authors: Idunn Brekke and Andreea Alecu**

**Corresponding author’s email: Idunn.brekke@fhi.no**

**Appendix A:** Predictors of maternal health problems, Average Marginal effects (AME) and t statistics in parentheses

|  | (1)  Musculoskeletal disorders  adjusted | (2)  Depression  adjusted | (3)  Anxiety  adjusted | (4)  Sleeping disorders  adjusted | (5)  Migraine  adjusted |
| --- | --- | --- | --- | --- | --- |
|  |  |  |  |  |  |
| **Child disability**, no disability (ref.) |  |  |  |  |  |
|  |  |  |  |  |  |
| Mild disability | 0.00927^***^ | 0.00260 | 0.00273 | 0.00204^**^ | 0.00156 |
|  | (3.99) | (1.68) | (1.77) | (2.86) | (1.67) |
|  |  |  |  |  |  |
| Complex disability | 0.00586^***^ | 0.00384^***^ | 0.00422^***^ | 0.000577 | 0.000893 |
|  | (4.07) | (3.60) | (3.79) | (1.64) | (1.59) |
|  |  |  |  |  |  |
| **Have had diagnosis prior to birth:** no (ref.) |  |  |  |  |  |
| Yes, at least one | 0.0546^***^ | 0.0276^***^ | 0.0188^***^ | 0.00268^***^ | 0.00812^***^ |
|  | (73.67) | (53.77) | (43.10) | (14.49) | (26.65) |
|  |  |  |  |  |  |
| **Year of education, mother** | -0.00124^***^ | -0.000160^***^ | -0.000454^***^ | -0.0000744^***^ | -0.0000448^**^ |
|  | (-30.01) | (-5.56) | (-16.23) | (-6.97) | (-2.69) |
|  |  |  |  |  |  |
| **Household income**, lowest quintile (ref.) |  |  |  |  |  |
|  |  |  |  |  |  |
| Second | 0.00711^***^ | 0.000104 | 0.000506 | -0.0000188 | 0.000810^***^ |
|  | (14.31) | (0.28) | (1.51) | (-0.13) | (4.29) |
|  |  |  |  |  |  |
| Third | 0.00729^***^ | -0.00224^***^ | -0.000373 | -0.00000979 | 0.000990^***^ |
|  | (14.50) | (-6.03) | (-1.10) | (-0.07) | (5.16) |
|  |  |  |  |  |  |
| Fourth | 0.00538^***^ | -0.00443^***^ | -0.00148^***^ | -0.000262 | 0.00132^***^ |
|  | (10.62) | (-11.93) | (-4.29) | (-1.83) | (6.62) |
|  |  |  |  |  |  |
| Highest | 0.00108^*^ | -0.00705^***^ | -0.00295^***^ | -0.000775^***^ | 0.00110^***^ |
|  | (2.12) | (-18.99) | (-8.35) | (-5.59) | (5.26) |
|  |  |  |  |  |  |
| **Number of children**, single child (ref.) |  |  |  |  |  |
|  |  |  |  |  |  |
| Two children | -0.00433^***^ | -0.00595^***^ | -0.00116^***^ | -0.000908^***^ | -0.000677^***^ |
|  | (-9.99) | (-18.37) | (-4.05) | (-7.37) | (-3.75) |
|  |  |  |  |  |  |
| Three or more children | -0.00642^***^ | -0.00766^***^ | -0.00244^***^ | -0.00140^***^ | -0.00112^***^ |
|  | (-13.84) | (-22.64) | (-7.98) | (-11.06) | (-5.87) |
|  |  |  |  |  |  |
| **Employment status**, non-employed (ref.) |  |  |  |  |  |
|  |  |  |  |  |  |
| Employed | -0.00648^***^ | -0.00739^***^ | -0.00840^***^ | -0.000726^***^ | -0.00125^***^ |
|  | (-15.08) | (-23.80) | (-26.90) | (-6.28) | (-7.16) |
| **Marital status,** Married/partner (ref.) |  |  |  |  |  |
|  |  |  |  |  |  |
| Divorced/Separated | 0.00377^***^ | 0.00996^***^ | 0.000791 | 0.000211 | 0.000580 |
|  | (5.04) | (15.15) | (1.44) | (1.19) | (1.85) |
|  |  |  |  |  |  |
| **Region of birth**, Norway (ref.) |  |  |  |  |  |
|  |  |  |  |  |  |
| Europe, North America, Australia | -0.00819^***^ | -0.00560^***^ | -0.00738^***^ | -0.000771^***^ | -0.00187^***^ |
|  | (-17.04) | (-17.20) | (-25.28) | (-6.58) | (-10.18) |
|  |  |  |  |  |  |
| Africa, Asia, Latin America | -0.00777^***^ | -0.00837^***^ | -0.0106^***^ | -0.00109^***^ | -0.00200^***^ |
|  | (-18.66) | (-33.85) | (-50.12) | (-11.82) | (-12.75) |
|  |  |  |  |  |  |
| Age at birth | 0.000744^***^ | -0.000221^***^ | -0.0000878^***^ | 0.0000874^***^ | -0.000126^***^ |
|  | (22.56) | (-9.01) | (-3.88) | (10.23) | (-8.99) |
|  |  |  |  |  |  |
| Child age | 0.00376^***^ | 0.00101^***^ | -0.0000221 | 0.000434^***^ | 0.000677^***^ |
|  | (67.17) | (25.88) | (-0.55) | (29.57) | (29.26) |
|  |  |  |  |  |  |
| **Child sex,** boys (ref) | 0 | 0 | 0 | 0 | 0 |
|  | (.) | (.) | (.) | (.) | (.) |
|  |  |  |  |  |  |
| Girls | 0.0000987 | -0.000979^***^ | -0.000493^*^ | -0.0000689 | -0.00000563 |
|  | (0.34) | (-4.82) | (-2.55) | (-0.91) | (-0.05) |
| N (person-years) | 1490196 | 1490196 | 1490196 | 1490196 | 1490196 |

^*^ *p* < 0.05, ^**^ *p* < 0.01, ^***^ *p* < 0.001
